# Review: Obesity and COVID-19: A Detrimental Intersection

**DOI:** 10.3389/fendo.2021.652639

**Published:** 2021-04-30

**Authors:** Maria Alessandra Gammone, Nicolantonio D’Orazio

**Affiliations:** Human and Clinical Nutrition Unit, Department of Medical, Oral and Biotechnological Sciences, University G. D’Annunzio, Chieti, Italy

**Keywords:** obesity, low-grade inflammation, COVID-19, adipose tissue, visceral fat

## Abstract

Obesity has been recognized as an independent risk factor for critical illness and major severity in subjects with coronavirus disease 2019 (COVID-19). The role of fat distribution, particularly visceral fat (often linked to metabolic abnormalities), is still unclear. The adipose tissue represents a direct source of cytokines responsible for the pathological modifications occurring within adipose tissue in obese subjects. Adipokines are a crucial connection between metabolism and immune system: their dysregulation in obesity contributes to chronic low-grade systemic inflammation and metabolic comorbidities. Therefore the increased amount of visceral fat can lead to a proinflammatory phenotypic shift. This review analyzes the interrelation between obesity and COVID-19 severity, as well as the cellular key players and molecular mechanisms implicated in adipose inflammation, investigating if adipose tissue can constitute a reservoir for viral spread, and contribute to immune activation and cytokines storm. Targeting the underlying molecular mechanisms might have therapeutic potential in the management of obesity-related complications in COVID-19 patients.

## Introduction

Obesity is more prevalent in developed countries, because of increased consumption of sugars, and saturated fats with low levels of fibers and antioxidant molecules. This diet can lead to enhancement of the innate immune system and delay of the adaptive immune system (interfering with T and B lymphocyte function), potentially via augmented oxidative stress (1). Obesity seems to increase the risk of both COVID-19 complications and mortality (2). Our perspective about COVID-19 is changing from a viral agent responsible for a respiratory disease toward a facilitator of complication and increased mortality in obese patients, with a dose-response curve, where higher body mass index result to be at most risk. This finding suggests a bidirectional relationship between COVID-19 and metabolic diseases: on the one hand, pre-existing metabolic diseases potentiate the severity of COVID-19, on the other hand, this viral infection exacerbate precedent metabolic frailty (3).

The amplified risk of obese patients to develop severe COVID-19 manifestations can depend on numerous factors, such as the chronic systemic phlogistic state, the decreased immune response, and even the adipose tissue itself, which represents a reservoir for the virus (4). Adipose tissue is also a fount of many proinflammatory mediators and hormones. High baseline serum C-Reactive Protein (PCR) and interleukin 6 (IL-6) levels, as well as hypoadiponectinemia and hyperleptinemia/leptin resistance (typical of obesity) elucidates the preexisting inflammatory microenvironment in obese patients, making them more susceptible to worse outcomes and even fatality. The perspective of obesity as a persistent low grade systemic inflammation is very interesting because of the multiple overlapping areas with the immune system. White adipose tissue produces adipokines, molecules that are involved in the etiopathogenesis of some major metabolic disorders such as dyslipidemia, diabetes, hypertension, and other cardiovascular disorders, which share a central role in the metabolic syndrome. Most adipokines with proinflammatory properties are overproduced, while others with anti-inflammatory or insulin-sensitizing properties (such as adiponectin) are reduced. This adiposity-linked dysregulation of adipokine secretion can contribute to obesity-related complications (5).

Two signaling pathways are activated by the proinflammatory cytokines in obesity: the nuclear factor-kB (NF-kB) and the c-Jun NH2-terminal kinase (JNK) pathway, which downregulates the anti-inflammatory transcription factors thus propagating inflammatory processes. Adipocyte hypertrophy is linked not only to mitochondrial dysfunction, DNA damage and cell death (6) but also to altered intracellular signaling: enlarged omental adipocytes resulted to be hyper-responsive to TNF-α with an increased constitutive NF-kB activity, thus leading to adipokine overproduction. Another crucial step in increased obesity-related inflammation is the massive infiltration of adipose tissue by phagocytosis mediators: monocyte chemoattractant protein-1 (MCP-1), whose circulating levels are elevated in obese, recruits monocytes and macrophages into the adipose tissue as well as into the arterial vessel wall. This process can contribute to cardiovascular events, which represent a frequent complication in COVID-19 patients (7).

Adipose tissue can regulate cardiac function through its endocrine effects, exerted not only by adipokines but also by lipokines (such as palmitoleate), batokines and exosomal miRNAs: a series of bioactive molecules contributing to the dialogue with metabolic tissues and influencing whole-body metabolism (8). Consequently, increased adiposity determines a dysregulation of endocrine functions, with consequent insulin resistance and higher cardiovascular risk. An impairment of immune system could also be present, due to the dysregulation of the factors produced by adipose tissue.

Obesity could also worsen the clinical outcome in COVID-19 also by respiratory compromise because of pulmonary restriction, impaired pulmonary perfusion, endothelial dysfunction and critical care management obstacles. In fact, obesity significantly interferes with respiratory function by reducing the expiratory reserve volume and the functional residual capacity. Further, the respiratory strength might be significantly reduced because of the ineffectiveness of the respiratory muscles, with the body fat distribution influencing the respiratory efficiency, due to the direct mechanical obstacle of visceral fat storage in the abdominal regions. These concurrent factors can lead to inspiratory overload and increase respiratory effort and oxygen request with a high likelihood of decompensation into respiratory failure and insufficiency.

## Visceral Fat: The Independent Risk Factor for COVID-19?

Preliminary data indicate that COVID-19 cases are mostly prevalent among obese subjects. Obesity constitutes a primary and independent risk factor for its complications in obese adults, where ectopic and visceral fat stores are the leading markers of such a risk. Even if growing evidence supports that obesity leads to an increased inflammation and fibrosis together with a decreased expression levels of the anti-inflammatory adiponectin also in the subcutaneous adipose tissue (8), visceral fat accumulation (the so-called “abdominal obesity” or “central obesity”), is usually more markedly characterized by an impaired profile of adipokines, with increased proinflammatory signals (9). In severe abdominal obesity, the adipokine profile is unbalanced in favor of leptin secretion and low-grade inflammation in spite of adiponectin. This deregulated adipokine profile links various metabolic disorders to inflammatory manifestations (9). This link has been recently evidenced in a cross-sectional analysis including 30 patients (aged 65.60 ±13.11 years) with COVID-19 diagnosis. An increase in visceral fat area by 10 cm^2^ at the level of the first lumbar vertebra was related to a 1.37-fold higher possibility of intensive care unit treatment and a 1.32-fold higher risk of invasive mechanical ventilation. One additional centimeter of waist circumference defines a more severe clinical course of COVID-19 with a 1.13-fold higher risk of intensive care unit treatment and a 1.25-fold higher possibility of assisted mechanical ventilation (10). Some authors also evidenced that computed tomography (CT) imaging of fatty liver and epicardial adipose tissue (EAT) were found in severely and critically ill young COVID-19 subjects, in comparison with patients experiencing milder disease (11). The increased risk of obese patients to develop severe COVID-19 cardiac and pulmonary damage could be referred not only to the mentioned factors (chronic phlogistic status in visceral obesity, unpaired immune response, and adipose tissue serving as a possible reservoir for the virus), but also to an additional mechanism: ectopic fat accumulation. Deng’s findings tried to extricate the complex physiopathology leading to COVID-19 organ injuries: they proposed that not simply obesity, but visceral fat is an independent risk factor for COVID-19 complications in young patients (12) because abdominal adiposity may promote and amplify the inflammatory answer (13, 14). Their study analyzed CT data of ectopic fat depots in young COVID-19 patients, such as intrahepatic and heart epicardial adipose tissue (EAT), which have been evidenced to play a role in COVID-19 myocardial phlogosis (15–17). EAT is an inflammatory depot with conspicuous macrophage infiltrates and proinflammatory cytokines, such as interleukin-6 (IL-6), which is secreted by numerous cells (monocytes, fibroblasts, endothelial cells, adipocytes), overexpressed in COVID-19 patients (18). Circulating levels and adipocyte release of IL-6 are augmented in obese individuals: specifically, visceral adipose tissue displayed to release three times more IL-6 than subcutaneous fat. Similarly, serum IL-6 levels resulted to be 50% higher in the portal vein than in the radial artery of obese patients; this portal vein IL-6 concentration resulted to be closely related to systemic C-reactive protein (CRP) levels. Visceral fat is a central site for IL-6 release, regulating the hepatic production of acute-phase reactants and providing a link between visceral adipose tissue and inflammation (19). EAT proinflammatory cytokines can reach out to the myocardium via vasa vasorum (since they share the same microcirculation) or through paracrine pathways (20), thus determining myocardial inflammation and concurring to cardio-respiratory failure. A profibrotic remodeling of extracellular matrix also plays a role in the pathogenesis of obesity complications, such as fibrosis of EAT.

The EAT is a metabolically active tissue releasing proatherogenic and proinflammatory molecules and leading to a low-grade inflammatory and pro-fibrotic environment. It was evidenced that EAT, through its secretion of adipo-fibrokines, such as Activin A (a member of the TGF-β family) and MMP8, may represent a complementary mechanism contributing to the genesis of myocardial fibrosis, implicated in the increased risk of arrhythmias in obese patients (21).

Peri-renal and liver fat infiltration may also play a pivotal role in severe COVID-19: visceral accumulation [often present in severely and critically ill young individuals with obesity (22)], may concur to hypoalbuminemia, insulin resistance, and hyperglycemia: all measurable predictors of COVID-19 complications, as recently evidenced (23). Another study (24) retrospectively examined EAT from CT scans of 41 patients, admitted for COVID-19 infection. Chest CT scan was performed on the admission day, investigating the presence of pulmonary embolism. EAT and subcutaneous adipose tissue densities were retrospectively obtained and defined as mean attenuation expressed in Hounsfield units (HU). EAT HU resulted significantly higher than subcutaneous adipose tissue HU (-95 HU versus -118, p<0.01); mean EAT thickness was 5.5 mm. EAT attenuation significantly augmented with the increase in COVID-19 severity. Subjects with more severe COVID-19 had more significant EAT attenuation, which was importantly linked to peripheral oxygen saturation (SpO2) and body temperature, and reflected inflammatory changes within the fat depot (25). Specifically, EAT showed imaging signs of augmented inflammation in patients with more severe COVID-19, directly increasing with COVID-19 severity. On the contrary, subcutaneous adipose tissue attenuation did not progress with the severity of COVID-19. Consequently, CT-measured EAT attenuation might play a diagnostic and prognostic role in COVID-19 patients with obesity (26), serving as a reliable (even if expensive) indicator of phlogosis in COVID-19 patients.

Hepatic and cardiac imaging could help to phenotype and stratify younger individuals with visceral obesity at higher risk of COVID-19 morbidity and mortality. Furthermore, visceral and ectopic fat can be targeted with lifestyle, such as weight loss and n-3 PUFA supplementation.

N-3 PUFAs can modulate the transcription of inflammatory genes through the regulation of key transcription factors (27), for example inhibiting nuclear factor kappa-light-chain-enhancer of activated B cells (NF-κB) and activating peroxisome proliferator-activated receptors-α/γ (PPARα/γ). N-3 PUFA-containing diets in severe ARDS showed to reduce the duration of mechanical ventilation, improved oxygenation and reduced stay in intensive care units (28).

A randomized controlled study (29) demonstrated that 1.5 g EPA and 1.0 g DHA 4 weeks daily supplementation can consistently decrease serum levels of TNF-α, IL-6,and IL-1β, suggesting n-3 PUFAs as an effective low-risk dietary intervention to mitigate the cytokine storm thus modulating inflammation. Similarly, the parenteral supplementation of 4g/die EPA and DHA in severe COVID-19 disease (30) inhibited cytokine production and mitigated the inflammatory response, thus attenuating the complex inflammatory state of pre-existing health conditions, such as obesity and elderly age, often associated with poorer clinical outcomes.

## Obesity and COVID-19 Pathogenesis

Although COVID-19 symptoms usually resolve in about 10 days, some patients develop respiratory insufficiency and become ventilator dependent (31–33) and need to be admitted to the intensive care units with respiratory failure: these patients are obese/overweight with extensive visceral fat in almost 90% cases (34). Visceral fat, lung tissue, and leptin production are closely interconnected. Leptin produced in visceral fat could contribute to deterioration in mechanical ventilation. In a cross-sectional study (35), COVID-19 patients displayed significantly increased leptin levels: the mean serum leptin level was 21.2 ug/L vs. 5.6 ug/L for COVID-19 patients and controls respectively. Within the same study, 90% of respiratory failure cases presented a body mass index over 25 kg/m^2^. Similarly, in the Seattle cohort individuals requiring mechanical ventilation presented a mean BMI of 33 kg/m^2^ (32, 33). An excessive adipose mass seems to contribute to the hyperinflammatory state, pulmonary phlogosis, and subsequent respiratory failure. A complex biological framework could explain these clinical observations. ACE2 receptor, which is a constituent of renin-angiotensin system (RAS), exerts a central role in the pathogenesis of SARS CoV2 COVID-19 infection. ACE2 receptors are expressed not only in the apical surfaces of well-differentiated ciliated cells, but also in endothelial cells, kidneys, pancreas, adrenals, and adipocytes. Intracellular invasion of COVID-19 is mediated by ACE2 receptor: the virus spike protein ‘‘S’’ is cleaved into S1 and S2 domains. The S1 fragment is internalized into the host cell through ACE2 receptor. The S2 domain is further cleaved further by the host cell (trans-membrane serine protease), (TMPRSS2), that determines membrane fusion with dissemination of the virus into the host (36). ACE2 receptor is present in the respiratory tract, lungs, and visceral fat; its expression is upregulated on alveolar epithelial cells and in visceral fat, particularly abundant in obese subjects. In addition, it has been very recently identified that the liver also expresses ACE2 and its priming protease TMPRS22, with obese patients with nonalcoholic steatoepatitis (NASH) showing increased hepatic expression of these critical viral entry points. In obese patients with NASH higher expressions of these genes have been evidenced, pointing that advanced stages of nonalcoholic fatty liver disease (NAFLD) could make subjects prone to COVID-19 infection (37). Specifically, expression of ACE2 receptor results to be upregulated because of obesity, air pollution, and smoking (38). This upregulation, more prominent in adipocytes of diabetic and/or obese subjects, could turn fat into a potential viral target and reservoir, thus explaining the reason why diabetes and overweight/obesity are dangerous comorbidities for COVID-19 infection, significantly augmenting the severity of the local pulmonic response (39). ACE2 is more expressed in visceral adipose tissue because of its antiobesity action in adipose tissue with stimulation of brown adipose tissue and browning of pre-existing white adipose tissue (40). The ACE2 utilization by the virus determines a local pulmonary inflammation due to ACE2-ATII disbalance. This may be enhanced by an increased leptin secretion (induced by COVID-19 infection of visceral adipose tissue): leptin receptors in the lungs are hyper-activated, thus enhancing local pulmonary inflammation. Since ACE2 reduces leptin levels through activation of the MrgD-receptor/c/Src/p38MAPK pathway, a compromised ACE2 function ends up in an additional elevation in leptin levels (41), resulting in a hyper-inflammatory local pulmonary answer embroiling local leptin receptors and local ACE2-ATII derangement. This disruption of the renin-angiontensin system by the virus impairs the energetic functions of these pathways during COVID-19 infection, leading to abnormalities in the inflammatory response through their influence on immune balance and cytokine generation (42). The major presence of ACE2 receptors in obese individuals is also due to a more abundant volume of adipose tissue. Weight loss achieved by bariatric surgery determines a significant reduction in adipose tissue volume, and also downregulates ACE 2 gene expression in the subcutaneous adipose tissue, which might also constitute a putative protective mechanism against severe COVID-19. Roux-en-Y gastric bypass was demonstrated to positively impact numerous comorbidities obesity-related (often linked to poorer COVID-19 outcomes), resulting independently associated with a reduced risk of mechanical ventilation and death in obese patients with COVID-19 (43).

## Endothelial Activation: Exacerbation of Inflammation

The chronic low-grade inflammatory state in obesity has been confirmed by high baseline CRP in overweight individuals (44, 45). A Korean multicentric study evaluated the correlation between high sensitivity-CRP and sarcopenic obesity: CRP resulted to be significantly higher in obese patients in comparison with the normal weight control group (46). This complex framework can explain respiratory failure in obese COVID-19 infected patients. Additionally, other obesity-related clinical features (such as tachypnea, minor lung capacity, reduced chest wall compliance, and aberrant respiratory muscle adaptations) can lead to severe respiratory failure requiring mechanical ventilation.

However, COVID-19 is associated not only with dysregulated inflammation but also with augmented coagulation and thrombotic accidents, which were extensively found in infected patients (47). In numerous ARDS patients, not only venous thromboembolism but also thrombocytopenia, renal failure, and disseminated intravascular coagulation have often been reported. In situ thrombi were found in pulmonic arteries and in other organs, including liver and kidneys, in subjects who died of COVID-19 (48).

A very recent study compared seven lungs from subjects dead from COVID-19 with seven lungs from subjects dead from H1N1-related ARDS infection and with age-matched uninfected control lungs after autopsy. The peripheral lungs from both COVID-19 and H1N1 presented a histologic pattern with a pervasive alveolar injury and perivascular T-cell infiltration. A COVID-19 peculiarity consisted of severe endothelialitis and damage, related to intracellular presence of virus and disrupted cell membranes, with diffuse alveolar capillary thrombosis and microangiopathy. Additionally, a greater amount of new vessel growth through a mechanism of intussusceptive vascular neoangiogenesis was reported (49). These autopsy findings raise the hypothesis that widespread endothelial hyper-activation in COVID-19 can trigger thrombotic events, with intra-alveolar deposits of hyper-activated leukocytes and fibrin contributing to pulmonary failure (50). The mechanism of this coagulopathy is not completely clear, however, dysregulated immune responses orchestrated by lymphocyte death, inflammation, hypoxia, and endothelial hyper-activation/injury are implicated (51). Specifically, endothelial cells could be hyper-activated by adipocyte secretory products; this activation results in intracellular signaling pathways leading to the generation of cell adhesion molecules, adipokines and proinflammatory cytokines, which address inflammatory cells to the endothelium and underlying tissues, stimulating them to become fully activated. Circulating levels of soluble intercellular (ICAM-1) and vascular (VCAM-1) adhesion molecules, as well as endothelial E-selectin result to be augmented in obese adults and reduced after weight loss. Similarly, incubation of human adipose tissue-derived endothelial cells with mature adipocytes resulted in the upregulation of endothelial cell adhesion molecules with augmented monocytes diapedesis. This process leads to firm adhesion of monocytes to the endothelium and increased diapedesis of macrophages through the endothelium junctions into the adipose tissue (52), so that adipokines and cell adhesion molecules can be directly involved in thrombosis. Additionally, both endothelium and adipose tissue generate plasminogen activator inhibitor-1, whose increased levels (mostly induced by TNF-α, thrombospondin-1 and oxidative stress) typical of obesity can determine hypofibrinolysis, thus configuring a pro-thrombotic state (53). Although the poorest outcomes in COVID-19 subjects certainly derive from multifactorial cofactors, thrombotic complications exert a pivotal role in their prognosis (54). The development of safe thromboprophylaxis strategies for these thrombotic complications goes through a full understanding of its pathophysiologic basis in COVID-19 patients.

## Metainflammation in Obese Patients: The Role of Leptin and TLR3 in Abnormal Viral Responses

Adipocyte dysfunction in visceral adipose tissue is closely related to low-grade chronic phlogosis, the so-called “metainflammation”, induced by both proinflammatory and hypoxic signals from adipocytes, which represents the starting point of metabolic diseases in obesity (55). The metainflammation often originates from the leptin-activated macrophages present in the white adipose tissue, which produce IL-6, TNF-α,and IL-1β, with the activation of the NF-kB pathway. This activation can be inhibited by adiponectine, whose effect is easily overwhelmed by this proinflammatory drive in cases of severe obesity (56).

Leptin is the leading adipokine, whose anorexigen activity modulates satiety and food intake; its blood levels are proportionate to the amount of white adipose tissue and BMI. Leptin is an essential pulsatile hormone, responsible for energy homeostasis and numerous neuroendocrine functions. It stimulates the migration of resident macrophages in the WAT and promotes their shift toward a proinflammatory profile, and unbalances lymphocyte Th profiles, by reducing regulatory T-cells and inducing Th17 polarization (57). The typical modern diet-induced obesity is characterized by hyperleptinemia (elevated levels of leptin) and resistance to the body weight reducing effects of leptin (58). Leptin resistance upsets the endothelial leptin signaling, predisposing to atherogenesis in obese subjects, and it is responsible for the proinflammatory microenvironment, often leading to cardiovascular complications (59, 60). Leptin also addresses hematopoiesis in the bone marrow toward granulocyte lines and promotes neutrophils survival: higher levels of neutrophils can be found in obese patients. This makes neutrophil recruitment more powerful in obese patients than in normal weight subjects during inflammatory processes (61). Additionally, this adipokine also induces vasodilatation by inhibiting angiotensin II-induced vasoconstriction and inducing nitric oxide release from vascular smooth muscle and endothelial cells, with leptin resistance blunting this vasodilatory effect (62).

However, obese patients not only suffer proinflammatory environments, but they also exhibit abnormal responses to viral infection. Specifically, patients with visceral adipose accumulation show not only a strong cytokine production, especially IL-6, IFN type I and III groups, interferon gamma-induced protein 10, but also a lower toll-like 3 receptor (TLR3) expression in adipocytes, muscle cells, and adipose tissue-resident macrophages (63). TLR3 belongs the toll-like receptor family, with a pivotal role in both microorganism recognition and stimulation of innate immune system. It identifies the double-stranded RNA of some viruses: upon recognition, TLR3 induces the production of type I IFN, which signals other cells to increase their antiviral defenses. Consequently, the reduced activation of TLR3 may exacerbate the metainflammation, thus contributing to the more severe septic states of obese patients with COVID-19. This suggests that their antiviral answer is less effective, but the overall phlogosis is higher than in normal weight subjects with viral infections. Additionally, individuals with metabolic abnormalities and visceral adipose accumulation could have a constitutional lower titer of angiotensin 1-7 (64), which limits the metainflammation as a guardrail, and a consequent higher inflammation; the inappropriate inflammatory response, added to the reduced activation of TLR3 in obese patients, can lead to unrestrained inflammation. This could contribute to the easier development of ARDS in obese patients.

## Conclusion

Obesity is a comorbidity that drives COVID-19 infected subjects toward a dangerous downhill slope. A low-grade chronic inflammation in obese people, as indicated by increased baseline serum levels of CRP, TNF-α, and IL-6 (positively correlated with BMI, waist circumference and visceral adipose tissue), is responsible for initiating the cytokine storm and can determine numerous underlying pathological situations and complications. Adipose tissue contains not only adipocytes (with significant intrinsic inflammatory properties) but also monocytes, macrophages, vascular components, and other cells contributing to inflammation. Additionally, adipocytes express numerous receptors, which are sensitized by infectious agents, thus activating cytokine-mediated signal transduction cascades and releasing proinflammatory cytokines and acute phase reactants. Up-regulation of ACE2 receptors in adipocytes is another reason why obese patients are more susceptible to infection with SARS-CoV 2 COVID-19 and progress into more critical forms of the disease. In this established interrelation between BMI-based obesity and severe course of COVID-19 disease, body fat distribution seems to be crucial, with visceral fat significantly raising the possibility of a more severe course of COVID-19: CT-based quantification of visceral adipose tissue and upper abdominal circumference can represent a simple promising strategy for risk assessment in COVID-19 infected subjects providing experimental proof of the association between visceral fat amount and COVID-19 severity: however it is not cost effective and expose the patients to radiations.

## Author Contributions

MG projected the manuscript and wrote the draft. ND’O supervised the process and contributed to editing. All authors contributed to the article and approved the submitted version.

## Conflict of Interest

The authors declare that the research was conducted in the absence of any commercial or financial relationships that could be constructed as a potential conflict of interest.
